# A Densely Connected Multi-Branch 3D Convolutional Neural Network for Motor Imagery EEG Decoding

**DOI:** 10.3390/brainsci11020197

**Published:** 2021-02-05

**Authors:** Tianjun Liu, Deling Yang

**Affiliations:** College of Engineering and Technology, Northeast Forestry University, Harbin 150040, China; 2019115320@nefu.edu.cn

**Keywords:** motor imagery (MI), electroencephalogram (EEG), dense connectivity, 3D convolutional neural network (3D CNN)

## Abstract

Motor imagery (MI) is a classical method of brain–computer interaction (BCI), in which electroencephalogram (EEG) signal features evoked by imaginary body movements are recognized, and relevant information is extracted. Recently, various deep-learning methods are being focused on in finding an easy-to-use EEG representation method that can preserve both temporal information and spatial information. To further utilize the spatial and temporal features of EEG signals, an improved 3D representation of the EEG and a densely connected multi-branch 3D convolutional neural network (dense M3D CNN) for MI classification are introduced in this paper. Specifically, as compared to the original 3D representation, a new padding method is proposed to pad the points without electrodes with the mean of all the EEG signals. Based on this new 3D presentation, a densely connected multi-branch 3D CNN with a novel dense connectivity is proposed for extracting the EEG signal features. Experiments were carried out on the WAY-EEG-GAL and BCI competition IV 2a datasets to verify the performance of this proposed method. The experimental results show that the proposed framework achieves a state-of-the-art performance that significantly outperforms the multi-branch 3D CNN framework, with a 6.208% improvement in the average accuracy for the BCI competition IV 2a datasets and 6.281% improvement in the average accuracy for the WAY-EEG-GAL datasets, with a smaller standard deviation. The results also prove the effectiveness and robustness of the method, along with validating its use in MI-classification tasks.

## 1. Introduction

Modern neurophysiological studies have proven that the power spectrum of some characteristic frequency components in electroencephalogram (EEG) signals can be altered by an actual body movement or imaginary motor movement. A reduction in the power spectral ratio is called event-related desynchronization (ERD), and an increase in the power spectral ratio is called event-related synchronization (ERS) [1,2]. The brain–computer interface (BCI), based on the ERS/ERD phenomenon, offers a way for communication between computers and the human brain by analyzing the electrical signals generated by the nervous system [3]. When people think of moving any of the body parts, this motor imagery-based system can trigger neuronal activity in some areas of the motor cortex related to the movements [4]. Brain signal recording methods are divided into invasive recording and noninvasive recording. Invasive recording is implanting the sampling electrode array into the brain through surgical craniotomy. Its advantages are high accuracy and less noise. Its disadvantages are that it cannot ensure the stability of the structure and function of the electrodes in the brain for a long time, because it is easy to cause immune responses and calluses, which lead to the decline or even disappearance of the EEG signal. On the contrary, a noninvasive recording method is achieved directly by attaching electrodes to the scalp to maintain good mechanical contact and conductivity. Compared with the invasive method, it is more simple and safe, and the experimental time is greatly shortened, but there is more noise, and a lot of training is needed. Because of their respective advantages, both methods are widely used [5]. The EEG signal is one of the widely used noninvasive electrical signals in BCIs. If these EEG signals are recorded and classified correctly, the classification results can be used to control auxiliary equipment such as robots and wheelchairs for motor-disabled people [6]. Thus, the classification of EEG signals can be effectively applied in BCIs. Note that many factors may affect the performance of BCIs, such as the health status of the subjects or the environment. Meng et al. [7] studied the effects of soft drinks and coffee under a resting state. The study is the first to show caffeine and sugar intake can affect brain–computer interface (BCI) performance.

On the basis of the ERS/ERD phenomenon, brain–computer interface (BCI) researchers have proposed a great number of methods for moto imagery (MI) classification. Various machine-learning algorithms are widely used in EEG signal classification. In previous studies, machine-learning methods such as dynamic connectivity analysis [8], frequency band analysis [9], continuous wavelet transform [10], and filter bank common spatial pattern (FBCSP) [11] have been widely proposed for EEG decoding. 

The general spatial pattern (CSP) algorithm and CSP-based methods are recognized as effective methods for feature extraction in brain–computer interfaces (BCIs). However, due to the inherent defects of the CSP objective function and the nonstationarity of the EEG signal, its corresponding features are not necessarily optimal in the feature space used by CSP. To address this issue, Jin et al. [12] designed a new feature-selection method by selecting features based on an improved objective function. In particular, some improvements were made in suppressing outliers and finding features with large interclass distances. On this basis, a fusion algorithm based on Dempster–Shafer theory was proposed. The experimental results show that the proposed method reduces the extra computational overhead and improves the performance of the MI-based BCI system significantly.

The performance of brain–computer interfaces (BCIs) based on motor imagery (MI) is vulnerable to noise and redundant information in multichannel electroencephalograms (EEGs) [13]. Jin et al. [14] proposed a correlation-based channel selection (CCS) method to select the channel with more relevant information. The purpose was to improve the performance of MI-based BCI classification. Furthermore, Jin et al. [15] also proposed a bispectrum-based channel selection (BCS) method for EEG classification based on BCIs, the sum of logarithmic amplitudes (SLA) and the first-order spectral moment (FOSM) features extracted from bispectrum analysis have been proposed for selecting EEG channels without redundant information. Their proposed algorithm can achieve better classification performance with fewer channels. 

Most of these methods artificially extract the time-frequency features from EEG signals and then combine these artificially extracted features into feature vectors, which are further used to classify the EEG signals in train classifiers such as support vector machine (SVM) [16] or decision tree [17]. Although CSP-based methods and other BCI frameworks have been successfully applied to MI classification, the architecture of these frameworks divides feature extraction and classification into two stages, which makes the feature-extraction model parameters and subsequent classifiers use different objective functions for training. Therefore, in the above-mentioned methods, the selection of the best filter band is subjective, and it depends primarily on the quality of the handmade features [18]. Thus, if the suboptimal frequency band is selected in the feature-extraction process, the classification performance might not be the best. In addition, for severe nonstationary EEG signals, the manual determination of the fixed frequency band may not be effective, so these methods may not be well extended to a larger population due to the heterogeneity among subjects.

Compared with the machine-learning framework, the deep-learning method [19] does not require the manual extraction of the feature. It embeds feature extraction and classification into a single end-to-end network; this end-to-end training method can well avoid the disadvantages of manual feature extraction in traditional machine learning. Recently, the application of deep learning in EEG classification tasks has become a research hotspot. To apply deep-learning methods to MI classification, EEG signals need to be represented in a processable form, which is a prerequisite to be satisfied. To meet this premise, EEG signals are often represented as a two-dimensional array, considering the number of sampling electrodes as the height and the time step as the width [20]. A typical method described in [21] is to represent EEG signals as 2D images by a short-time Fourier transform (STFT) method. 

However, this two-dimensional representation cannot keep the spatial information of the EEG, and the correlation between the adjacent electrodes cannot be reflected in the two-dimensional array, which results in the unsatisfactory classification performance of EEG coding. To address the shortcomings of the above two-dimensional representation methods and to obtain better performance, some more-dimensional representation methods were introduced. Zhao et al. [22] proposed a new 3D representation method for EEG signals, which not only retains the spatial features but also retains the temporal features. Based on that, they proposed a multi-branch 3D convolutional neural network (CNN) with only three convolution layers in each branch to automatically extract the MI-related features. However, certain issues are yet to be answered in this research. Although such a 3D representation and 3D CNN can maximize the retention of time and space features, it also introduces some invalid features in the 3D representation because of the padding of the no-electrode point with 0. On the other hand, the results in [23,24] indicate that adapting deep architecture can improve the training effect; a deeper model means better nonlinear expression ability, can learn more complex information, and can fit more complex feature input, but the multi-branch 3D CNN possesses only three convolution layers, which is too shallow for extracting the EEG signal features; meanwhile, simply increasing the depth of the network will easily lead to an overfitting problem [24].

To address these problems, a new 3D presentation method has been proposed in this study, which preserves both spatial and temporal features similar to the 3D presentation in [22], and at the same time, it fully utilizes the features of EEG signals in the 3D presentation. Based on this improved 3D presentation, a deeper and novel end-to-end deep-learning architecture called densely connected multi-branch 3D CNN (dense M3D CNN) is proposed to extract the EEG signal features and complete classification tasks; differently from simply increasing the depth of the network, our method can prevent the overfitting problem because of its dense connectivity. One of the primary contributions of the proposed framework is that the improved 3D presentation completely utilizes the features of the EEG signal and partially improves the classification accuracy. Another contribution of the proposed method is that new dense connectivity is employed to design the competitive densely connected multi-branch 3D CNN, which eventually enables the network to effectively learn the temporal and spatial information for classification. The most advanced performance on WAY-EEG-GAL and BCI competition IV-2a datasets has been achieved through this study. The classification performance was significantly improved on two public datasets.

## 2. Methods

In the following sections, we will first describe the improved 3D representation of the EEG and then describe the overall architecture of the multi-branch dense 3D CNN block and structure of the densely connected multi-branch 3D CNN.

### 2.1. Improved 3D Representation of EEG

Zhao et al. [22] designed a three-dimensional model of the EEG. Firstly, according to the distribution of the sampling electrodes, the EEG signal is converted into a two-dimensional array, and then, the points without electrodes are filled with 0. After that, this 2D array is expanded to a 3D array by using the temporal information of the EEG signals. However, such a padding method introduced many invalid features in the 3D representation and did not permit the efficient utilization of the 3D presentation of all the electrode signals. When it comes to comparing the representation of EEG signals in this paper with that of the padding method in [22], the points without electrodes here are padded with the mean of all the EEG signals.

Note that for the BCI competition IV-2a, in a 7 × 7 × 240 3D presentation, “7 × 7” represents the spatial distribution of the electrodes, which is determined by the number and distribution of the sampling electrodes retaining the spatial information of the EEG, and “240” means the sampling points of the EEG signal, which are determined by our cropped strategy (introduced in the following section) and retain the temporal information of the EEG signal.

**Figure 1:**
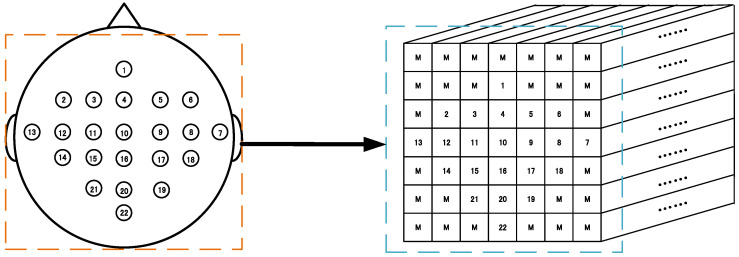
3D representation of the electroencephalogram (EEG) signal for the BCI competition IV-2a dataset. Left: sampling electrode’s spatial distribution. Right: 3D representation (7 × 7 × 240 array) of EEG. M means the mean of all EEG signals. (1–22 electrodes.)

**Figure 2:**
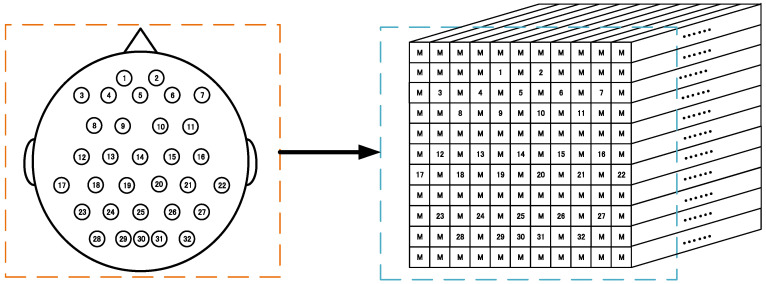
EEG signal 3D representation for the WAY-EEG-GAL datasets. Left: sampling electrodes’ spatial distribution. Right: 3D representation (11 × 11 × 480 array) of EEG. M means the mean of all EEG signals. (1–32 electrodes.)

This padding method is easy to handle and can make full use of the 3D presentation of all the EEG signals. Figure 1 schematically demonstrates the 3D representation (7 × 7 × 240 array) according to these 22 sampling electrodes’ locations in a 10–20 system based on the BCI competition IV-2a. Similarly, Figure 2 schematically shows the 3D representation (11 × 11 × 480 array) according to these 32 sampling electrodes based on the WAY-EEG-GAL dataset. It can be observed that this representation method not only completely retains the temporal information of the EEG signals but also retains the spatial information of the sampling electrode’s distribution to a certain extent, at the same time resulting in the complete utilization of the 3D presentation of all the electrode signals. 

### 2.2. The Densely Connected Multi-Branch 3D CNN

Based on the 3D representation, a densely connected multi-branch 3D CNN was introduced in this study for MI classification. In this section, the layout of the densely connected multi-branch 3D CNN is introduced, including the multi-branch dense 3D CNN block and the overall structure of the proposed dense M3D CNN. Later, the specific configuration is described.

#### Network Overview

Huang et al. [25] proposed a novel convolutional network architecture (dense convolutional network) wherein the dense connectivity improves the back propagation of the gradient, making the network easier to train, thereby improving the classification effect. Based on this, similar dense connectivity in our deep 3D CNN was introduced.

The multi-branch dense 3D CNN block: Inspired by [22], a dense multi-branch 3D CNN was designed as a dense 3D CNN block with three different receptive field networks to extract the EEG signals’ features. Furthermore, a dense connection in the three networks was observed. These were further combined into a dense block referred to as the dense multi-branch 3D CNN block.

**Figure 3:**
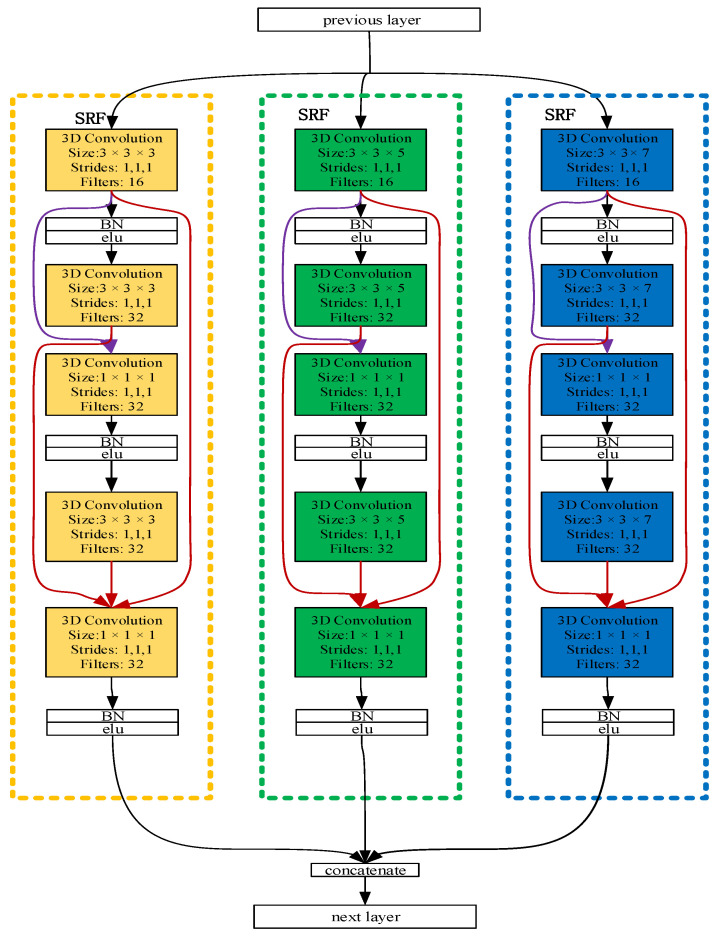
The densely connected multi-branch 3D convolutional neural network (dense multi-branch 3D CNN) block. Three branch networks are framed by three dashed boxes with different colors, and the input of different convolution layers is distinguished by connecting lines of different colors.

Figure 3 illustrates the overall architecture of the dense multi-branch 3D CNN block. The feature maps from the previous layer as input to the current layer. After convoluting through a 3D convolution layer, it is connected to the feature maps generated by all the previous 3D convolution layers. The bottleneck layers (1 × 1 × 1 3D convolutional layers) are introduced after a concatenation operation to reduce the input feature maps and improve the computational efficiency.

**Figure 4:**
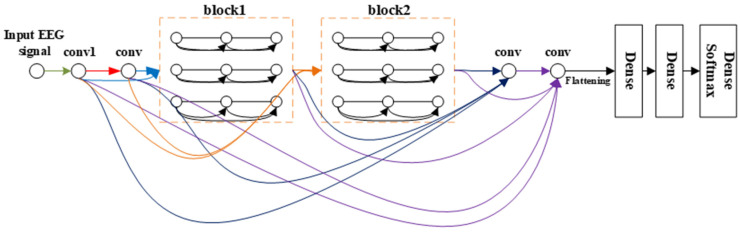
The overall structure of densely connected multi-branch 3D CNN, where the input of different convs is distinguished by connecting lines of different colors. Conv means 3 × 3 × 3 3D convolution layer + bottleneck layers + batch normalization (BN) + ELU.

**Overall structure.**Figure 4 schematically depicts the architecture of the resulting densely connected multi-branch 3D CNN. Consequently, the layer concatenates the feature maps produced by all the preceding layers. Unlike in the method in an earlier study [25], the connection occurs after the convolution layer; i.e., the connection develops between the feature maps generated by the current convolution layer and those produced by all the previous convolution layers, as shown in Figure 3. With X_0_, X_1_ . . . . . . X_Ɩ−1_ as input:

(1) $$X_{l}=H_{l}([X_{0},.\ldots \ldots X_{l-1}])$$

where [X_0_, X_1_ … X_Ɩ−1_] means the concatenation of the feature maps generated by all the previous 3 × 3 × 3 convolution layers: 0, ..., Ɩ − 1. This network is referred to as the densely connected 3D convolutional neural network due to its dense connectivity. Just as in the dense connection method of the dense multi-branch 3D CNN block in Figure 3, in (1), H_Ɩ_ is a composite function with three continuous operations: bottleneck layers (a 1 × 1 × 1 3D convolution layer) to reduce the number of input feature maps and batch normalization (BN) [26], followed by an ELU, an excellent activation function. Besides, the depth can be easily adjusted by changing the number of dense blocks in the model.

**Figure 5:**
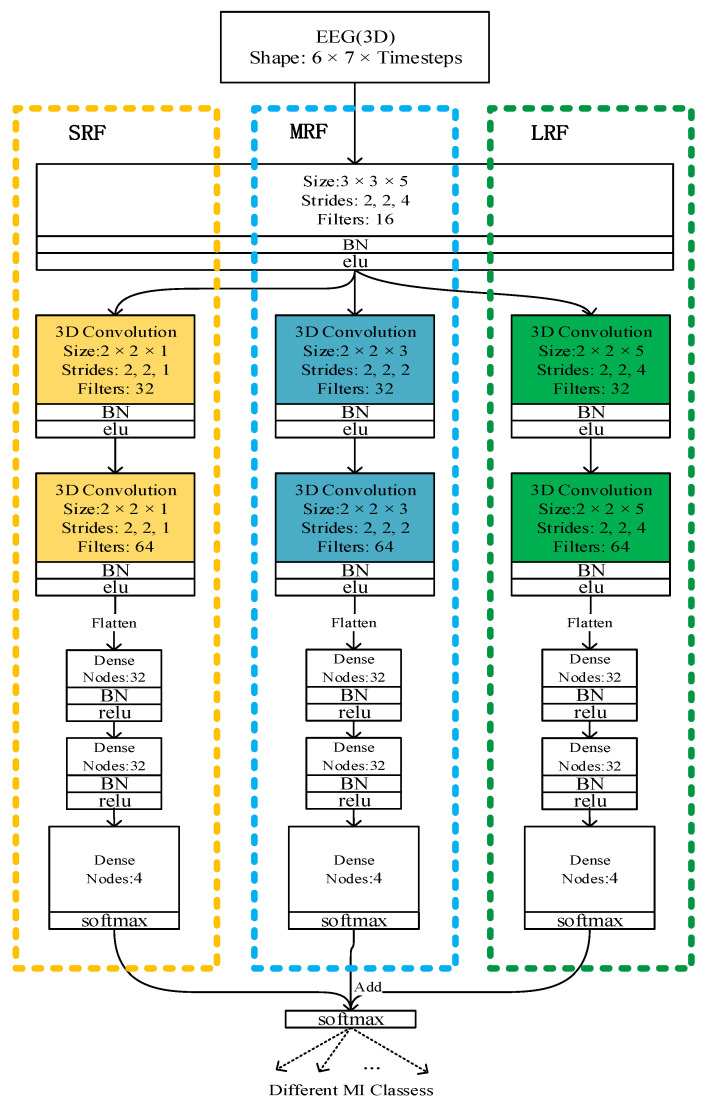
The multi-branch CNN architecture.

From the comparison between Figure 4 and Figure 5, we can see that our framework is much deeper than that of [22]. The multi-branch 3D CNN possesses only three convolution layers, which is too shallow for extracting the EEG signal features, but our network has 10 convolution layers with dense connectivity, which can better extract the features of the EEG signal, so as to obtain a better classification effect.

### 2.3. Details about Dense 3D CNN

As shown in Figure 4 and Table 1, there are 10 convolutional layers (which include six convolution layers in the dense block), which are all connected with dense connectivity in three dense layers. Meanwhile, a dropout method is introduced after the two dense layers to alleviate the overfitting problem. The dropout percentages are 0.5 (the optimal dropout percentages were obtained through many experiments with different dropout percentages ranging from 0.3 to 0.7 with an interval of 0.1). Note that, compared to the framework proposed by Zhao et al. [22], this method significantly improves the depth of the network to effectively extract the features of the EEG signal, along with improving the classification performance of the network. Inspired by the structure of VGG [23], the classical small convolution filter (3 × 3 × 3) was chosen to reduce the parameters and increase the network depth.

### 2.4. Training and Testing Strategy

Cropped training has been applied to the image recognition field for increasing the training data and improving the training effect [24,27]. In terms of EEG processing, cropped training is also widely preferred [20,28]. The cropped strategy on BCI IV 2a datasets [22] was adopted here. A cropped training approach for EEG 3D representation was adopted by sliding a 3D window that covers all the electrodes on each EEG data trial along the time dimension with a data stride of 1. The 3D window size on the time dimension is related to the sampling rate of the EEG data and the characteristics of the specific task; for the BCI competition IV 2a dataset, since the sampling frequency is 250 Hz, the 3D window size was 240. Through this approach, the cropped strategy with a temporal size of 240 would generate 74 cropped data for a given original trial with 313 timesteps.

For WAY-EEG-GAL datasets, a new data cropped strategy has also been proposed, which will be introduced later.

When it comes to network optimization, similar to in earlier work [25], all the weights, as well as the initial value, were initialized using the normalized initialization method in [29], and the learning rate was 0.01. The negative log-likelihood cost was adopted as the optimization criterion, and the optimization method used ADAM with the default parameter values described in [30]. In the training process, if the cost did not reduce within 20 epochs, the training was stopped, and the network weight with the lowest cost was restored from the epoch.

In the process of testing, a clipping strategy was used to improve the testing accuracy, too. Considering the real-time performance of the 3D CNN network in the test process, we set the data stride parameter of the clipping strategy to 5, which reduced the calculation time.

## 3. Experiments and Results

### 3.1. EEG Data

The proposed methods were evaluated on the WAY-EEG-GAL and the BCI competition IV 2a datasets. 

The BCI competition IV 2a dataset consists of EEG data from nine subjects; 22 Ag/AgCl electrodes were used to record the EEG signals. Each subject recorded two sessions on different days, and the recorded signals were sampled at 250 Hz. The recorded signals were sampled at 250 Hz and bandpass filtered between 0.5 and 100 Hz. A single run consisted of 48 trials, which yielded 288 trials per session. The duration of each trial consisted of a fixed period of 2 s and a reminder period of 1.25 s, followed by a period of 4 s of motor imagery. More details on the datasets are available in [31]. 

In the presented study, a 1.25 s period of EEG data were chosen as the experimental data, after the visual cue in each trial. These were further represented as 3D representations without any preprocessing. The sampling frequency was 250 Hz, so 313 sampling points could be generated in 1.25 s of sampling time. It can be concluded from the results of [22] that for the EEG signal with a 250 Hz sampling frequency, the EEG signal with 240 sampling points covered the features related to motor imagery.

Additionally, the WAY-EEG-GAL dataset is not only the first but also the only published dataset of EEG signals related to different stages of action identification tasks obtained from 12 subjects. The EEG data in this dataset consist of all the EEG signals in the whole process of the experimental paradigm. In the aspect of EEG signal recording, 32 EEG sampling electrodes were used, which meet the international 10–20 standard. In the process of each subexperiment, the EEG signals were continuously sampled by the EEG sampling electrode, with a sampling frequency of 500 Hz. In terms of the time-point recording of experimental data, the dataset provides 43 time-point pieces of information: the start time of each subexperiment, the time when the indicator LED lit up, and the time when the indicator LED light went out. Through such time-point information, the brainwave signal data could be mapped with different events, one by one. This time-point information was placed in the human joints or moved from sensors on the surface of an animal. A complete description of the grasp-and-lift experiment is available in [32].

As the sampling frequency of these data is 500 Hz, the time steps were adjusted to 480 sampling points to match the previous research results.

The experimental results in [19] prove that cropped training has better classification performance than trial-wise training. For the BCI competition IV 2a dataset, a cropped training method for EEG signals was adopted from an earlier study [22], such that the 3D window covered all the sampling electrodes along the temporal data with a stride of 1, by sliding a 3D window. A clipping strategy with a time size of 240 generates 74 clipping data, as given in an original EEG signal with 313 time steps. In this study, the same cropped strategy was used as in [22] to expand the training and test datasets for the BCI competition IV 2a dataset.

For the evaluations using cross-validation, the training and testing datasets of the BCI IV dataset 2a were combined and then randomly divided into ten subsets of equal size, out of which nine subsets were used as training data, and one of them was used as the validation data in each run. 

While dealing with the WAY-EEG-GAL datasets, considering the continuity between the stages of the action, four-class MI-classification experiments were transferred to three continuous binary classification experiments; at the same time, due to the reduction in classification classes, the numbers of nodes in the last dense layers were reduced to 2; for this experiment, our training strategy was the same as the one for the BCI competition IV 2a dataset. A cropped method similar to that mentioned earlier was used [22]. Cropping was performed with a temporal length of 480. Thus, the cropped strategy generated some cropped data, as shown in Table 2. Note that the achieved numbers of training data for different stages were unbalanced.

In order to balance these training datasets, another data-cropping method was proposed. This adjustment method follows the principle of keeping the data number of each class at around 6000 or less. There are two distinct cropping methods for different situations. When clipping with a cropped step of 1, if the number of cropped data for some classes is still less than 6000, the data are cropped with 1, and based on the number of these data, another class is cropped. Thus, the cropped step size can be calculated as:

(2) $$\mathrm{step}=\frac{num\_c1}{num\_c2}$$

where *num_c*1 is the number of data in a class with more than 6000 data and *num_c*2 is the number of data of another class with fewer than 6000 data.

If the number of data for both the classes is more than 6000 when clipped with a cropped step of 1, the number of cropped data is set to around 6000 by clipping them with steps such as (3) and (4).

(3) $$\mathrm{step}1=\frac{num\_c1}{6000}$$

(4) $$\mathrm{step}2=\frac{num\_c2}{6000}$$

where *num_c*1 is the number of the class 1 data and *num_c*2 is the number of the other class’ data.

Note that this cropped method can ensure that the training data of each class are almost equal but not completely equal. The purpose of this method is to balance different types of EEG data for improving the classification effect of the model. For the evaluations using the cross-validation of the WAY-EEG-GAL datasets, the training and testing datasets were combined and then randomly divided into nine subsets of equal size, out of which eight subsets were used as training data and a single subset was used as the validation data in each run. 

The average value of each cropped 3D array was subtracted from two datasets, and the training datasets were shuffled before each training epoch. 

### 3.2. Overall Comparison

The framework described in an earlier study [22] was compared with our proposed three dense M3D CNNs with different depths using the BCI competition IV-2a and the WAY-EEG-GAL datasets.

The multi-branch 3D CNN is a deep-learning framework with a three-branch 3D CNN, where each branch has a distinct receptive field. Based on previous studies, the multi-branch 3D CNN is considered to be a state-of-the-art classification method for the BCI IV 2a. 

The multi-branch 3D CNN was compared with the proposed three kinds of densely connected 3D CNNs with different depths, namely, the dense M3D CNN1, dense M3D CNN2, and dense M3D CNN3, which contain one, two, and three dense blocks, respectively.

Table 3 and Table 4 summarize the classification accuracies exhibited by the multi-branch 3D CNN and the proposed three dense 3D CNNs with different depths for both the BCI IV 2a and the WAY-EEG-GAL datasets, respectively. From Table 3, it can be observed that for the BCI IV 2a datasets, the multi-branch 3D CNN achieved a 75.015% average classification accuracy for all the subjects.

The average accuracy of the dense M3D CNN1, dense M3D CNN2, and dense M3D CNN3 reached 80.060, 81.223, and 79.108%, respectively, all higher than that for the multi-branch 3D CNN, with a *p*-value < 0.01. The dense M3D CNN2 achieved the highest accuracy of 81.223% and the lowest standard deviation of 6.845%, which means the dense M3D CNN2 has the best performance and excellent robustness for different subjects. 

To further certify the classification ability of the proposed method, the performance of the multi-branch 3D CNN was evaluated for the proposed methods on the WAY-EEG-GAL datasets. The results in Table 4 are the average classification accuracies for 12 subjects. Table 4 clearly illustrates the experimental results for the dense 3D CNN used on the WAY-EEG-GAL datasets, which is encouraging, as the dense 3D CNN with three dense blocks (dense M3D CNN3) achieved a significantly higher average accuracy of 76.960% than the multi-branch 3D CNN. Moreover, the average accuracy increased as the network depth became deeper. The average accuracies of the dense M3D CNN1 and dense M3D CNN2 reached 76.428 and 76.960%, respectively, which are also remarkably more competitive than that of the multi-branch 3D CNN. These experimental results verify the extraordinary performance of the proposed densely connected multi-branch 3D CNN with an improved 3D presentation method for decoding the EEG signals of motor imagery.

### 3.3. Influence of New 3D Presentation

Based on the overall comparison stated above, the dense 3D CNN with new 3D presentation is significantly more competitive than the multi-branch 3D CNN. In this section, we describe how the proposed dense M3D CNN2 and multi-branch 3D CNN were trained with the 3D presentation proposed earlier [22] and the currently proposed 3D presentation, respectively, on the BCI IV 2a datasets. 

The experimental results in Table 5 prove that our proposed 3D presentation improves the performance in EEG decoding. The average accuracy of the training with the proposed 3D presentation was 1.86 and 0.285% higher, respectively, for two different networks than that of the training with the old 3D presentation in [22]. Simultaneous 0.217 and 0.137% reductions in standard deviation were also observed for the different subjects, respectively. The proposed 3D presentation pads the points without electrodes with the mean of all the EEG signals, which also contain the features of all the electrode signals, instead of zero. This means, compared to the 3D presentation described in [22], the proposed 3D presentation not only retains the entire temporal information of the EEG and the sampling electrodes’ spatial distribution information but also makes full utilization of the 3D presentation of all the electrode signals to improve the effectiveness and robustness of the framework.

### 3.4. Influence of Dense Connectivity

Huang’s research [25] found that dense connectivity has several impressive advantages such as encouraging feature reuse, enhancing feature propagation, and effectively alleviating the vanishing-gradient problem. To demonstrate the efficacy of dense connectivity, a 3D CNN was designed without dense connection (namely, the M3D CNN2), which had the same depth and parameters as the dense M3D CNN2 described in Section B. It was further compared with the proposed network for the BCI IV 2a datasets. 

Table 6 shows the detailed results for both the networks. The average accuracies of the 3D CNN and proposed dense 3D CNN reached 77.114 and 81.223%, respectively. Compared to the 3D CNN without dense connectivity, the classification accuracy of the proposed network for subject 2, subject 3, and subject 5 was significantly improved to 6.863, 5.556, and 5.135%, respectively. These experimental results prove that our network has better classification effects for all the subjects, with smaller standard deviations, thereby indicating that the dense M3D CNN2 is more effective and robust owing to its dense connectivity.

### 3.5. Different Numbers of Branches in Dense Block

For the experiments mentioned earlier, the results were compared for different depths of the network, indicating that the classification effect is the best for a densely connected multi-branch 3D CNN with two dense blocks. The experimental results in an earlier study [22] confirm that compared to the results for three single-branch 3D CNNs, the results for the multi-branch 3D CNN show better performance than the other three single-branch networks for nine subjects. In general, the multi-branch 3D CNN achieved higher average classification accuracies and lower standard deviations for different subjects, which proves that the multi-branch architecture is more effective and robust than the single-branch architecture. To further explore the influence of the number of branches in the dense block on the classification accuracy, a set of experiments was carried out on three networks with different numbers of branches, which were composed of SRF, SRF and MRF, and a multi-branch 3D CNN dense block. 

Table 7 summarizes the classification accuracies derived for the three networks with different numbers of branches in the dense blocks. Particularly, the * indicates that there is one branch in the dense block, and the ** suggests the presence of two branches.

The results in Table 7 suggest that the classification performance of the dense 3D CNN improves with the number of branches in the dense block, with the average accuracies of the dense 3D CNN *, dense 3D CNN **, and dense M3D CNN reaching 78.895, 79.526, and 81.223%, respectively.

Meanwhile, the standard deviation for the network with three branches was only 6.845%, which is lower than that for the other methods. Comparing the three different networks, it can be concluded that the more-branch architecture can significantly outperform fewer-branch architectures for all the subjects. This indicates that the more branches the network of the model has, the better the model can complete the classification task.

**Figure 6:**
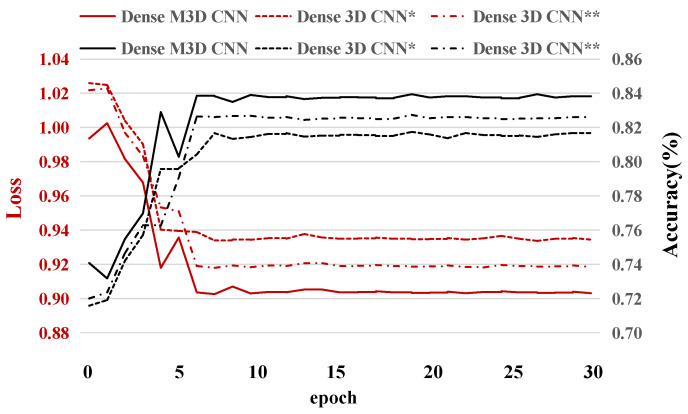
The test accuracies and test losses for subject 1 during thirty epochs. The * indicates that there is one branch in the dense block, and the ** suggests the presence of two branches.

Visualizing the convergence process of network training can help us to understand the performance of the network more intuitively. In this subsection, three different 3D CNN networks’ (the dense 3D CNN *, dense 3D CNN **, and dense M3D CNN) accuracies and losses for subject 1 (Because the results for the other subjects were very similar, we analyzed them with subject 1 as the representative.) for the experimental validation dataset for thirty training epochs are described. From Figure 6, we can see two interesting phenomena. First, all the models (including the dense 3D CNN * and dense 3D CNN **) converged, and the loss values and accuracies were in a stable state from the sixth training epoch, which means that our network has a fast learning ability and can prevent overfitting well. According to this phenomenon, in our experiment, we only trained fifteen epochs for all the networks on all the subjects. Second, the performance of the dense M3D CNN was slightly better than that of the other two networks, which was due to the combination of more branches with different filter sizes, which can better extract the features of EEG signals from different dimensions.

Overall, the results of the experiments demonstrate that, owing to the dense connectivity, our proposed dense M3D CNN performs well during the training process without any obvious signs of overfitting, which shows its resistance to overfitting, achieving improved performance. 

### 3.6. Different Electrode-Selection Modes and Comparison with the State of the Art

**Figure 7:**
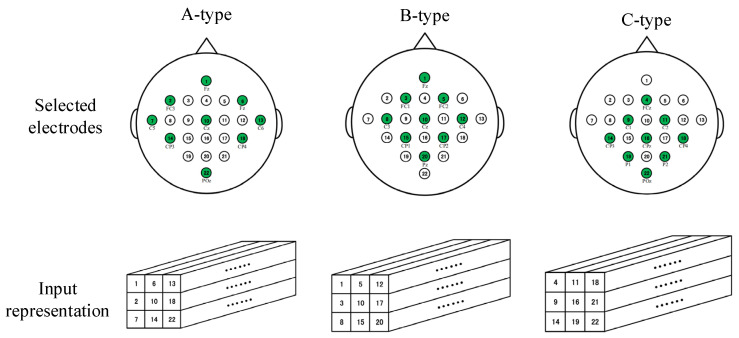
The selection modes of three sampling electrodes and the corresponding input representation methods. The circles filled with green indicate the selected electrodes.

Research shows that BCI systems with fewer electrodes can also achieve good performance [22], and the real-time performance and convenience of full-set EEG sampling electrodes are not as good as those of sets of fewer sampling electrodes. In this section, we describe the adoption of four optional electrode-sampling modes, named A-type, B-type, and C-type, as shown in Figure 7. At the same time, the parameters of the dense M3D CNN were adjusted (The sizes of all the convolution layers except the first share layer were changed to 2 × 2 × 3.) for different kinds of electrode-selection mode. 

The performance of three types of subset sampling electrode-selection modes, full-set EEG sampling electrode modes, and three state-of-the-art MI-classification methods were compared. The classification results were calculated using the testing dataset of each subject using the weights trained on the training dataset of the corresponding subject.

Here, Cohen’s kappa coefficient [33] was used to evaluate the performance of the different networks. The kappa values reported in Table 8 were all averaged over 50 results using different model initializations. The kappa value is defined as in (5), where $P_{0}$ is the proportion of observed agreements and $P_{e}$ is the probability that the agreement is due to chance.

(5) $$Kappa=\frac{P_{0}-P_{e}}{1-P_{e}}$$

We briefly introduce three state-of-the-art algorithms.

FBCSP: FBCSP [11] is a two-stage method. Firstly, a group of bandpass filters and the CSP algorithm are used to extract the optimal spatial features from a specific frequency band, and then, the classifier is trained to classify the extracted features.

C2CM: C2CM [34] first uses FBCSP as a data preprocessing method, and then uses the CNN to extract features. The performance of this method is better than that of FBCSP, but the trouble is changing the parameters according to different objects.

Multi-branch 3D CNN: The multi-branch 3D CNN [22] is a deep-learning framework with a three-branch 3D CNN, where each branch has a distinct receptive field. Based on previous studies, the multi-branch 3D CNN is considered to be a state-of-the-art classification method for the BCI IV 2a. 

Table 8 summarizes the classification accuracies derived for the FBCSP, C2CM, multi-branch 3D CNN, and dense M3D CNN with full-set EEG sampling electrodes and A-type, B-type, and C-type for the BCI IV 2a datasets, respectively.

On the one hand, by comparing the results for these networks, we can see, intuitively, the advantages of our network with full-set EEG sampling electrodes; the kappa value of our network is much higher than the values of all the other networks. Especially, compared with C2CM, which has the highest kappa value as far as we know, the kappa value of our proposed network is 0.21, which is 0.062 higher than that of the C2CM, with a 0.083 decrease in variance. Compared with the other two algorithms, the FBCSP [11] and multi-branch 3D CNN [22], our method is more significant and has a better classification effect; although the kappa variance of our network is slightly larger than that of the multi-branch 3D CNN [22], the robustness of our network is still at a high level, which means our framework performs significantly more robustly for different subjects.

On the other hand, our proposed dense M3D CNN with fewer sampling electrodes also achieved a state-of-the-art classification effect. From Table 8, we can see that, for the BCI IV 2a dataset, the dense M3D CNN with the A-type, B-type, and C-type sampling methods proposed in this study achieved 0.641, 0.604, and 0.608 kappa values, respectively, for nine subjects, all higher than the 0.571 kappa value of FBCSP. This shows that the proposed method is competent for EEG signal classification even if fewer sampling electrodes are used.

### 3.7. Time Consumption

Compared with the 3D CNN, our network effectively deepens the depth of the network and introduces dense connections, which makes the classification effect of the network significantly improved, but it also inevitably increases the complexity of the network, resulting in an increase in network parameters and training time. In the practical application of BCI systems, the classification accuracy and time consumption are two factors to be considered. The experimental results from [3] show that a BCI system with a response within 1 s is very suitable for real-time applications. In this section, we describe how, to evaluate the practical performance of a BCI system that was based on the proposed framework, we performed the training of the dense M3D CNN with different sampling electrode modes (full-set sampling electrodes, and A-type, B-type, and C-type) on a GeForce GTX 1060Ti GPU with 6 GB of memory. In detail, the time for fifteen training epochs for subjects one to nine for the whole training dataset (about twenty-one thousand samples) was recorded as the training time, and then, the average value of nine training times was taken. Similarly, the testing time for subjects one to nine for the whole testing dataset (about twenty thousand samples) was obtained in this way. The average training time and testing time for all the subjects was calculated for the BCI IV 2a dataset, and the results for the training and testing times with the different methods are listed in Table 9. The results show that on the one hand, the training time and testing time of the dense M3D CNN with full-set sampling electrodes were 33 min and 5 s and 21 s, longer than those of the A-type, B-type, and C-type. This means that fewer electrodes can significantly make the training time less, but also inevitably make the classification effect worse. On the other hand, though our dense M3D CNN suffers from a high training time, its fast prediction speed (1.04 × 10^−3^ s, 21 s for about twenty samples) and high prediction accuracy makes it more competitive for the implementation of online BCI systems or other clinical applications.

## 4. Discussion

### 4.1. Visualization of Intermediate Features

Visualizing the intermediate features of our proposed network can help us to understand how the network learns and why it achieved such high-quality results. In this section, we describe how we visualized the intermediate features of our proposed network with the A-type input mentioned above. The network was trained on the training dataset of subject 1 in advance.

The A-type EEG signal of subject 1 was selected as the sample input because this input has fewer sampling electrodes, and the corresponding network also achieves a relatively acceptable performance level, which helps in obtaining more concise and easier-to-understand results.

**Figure 8:**
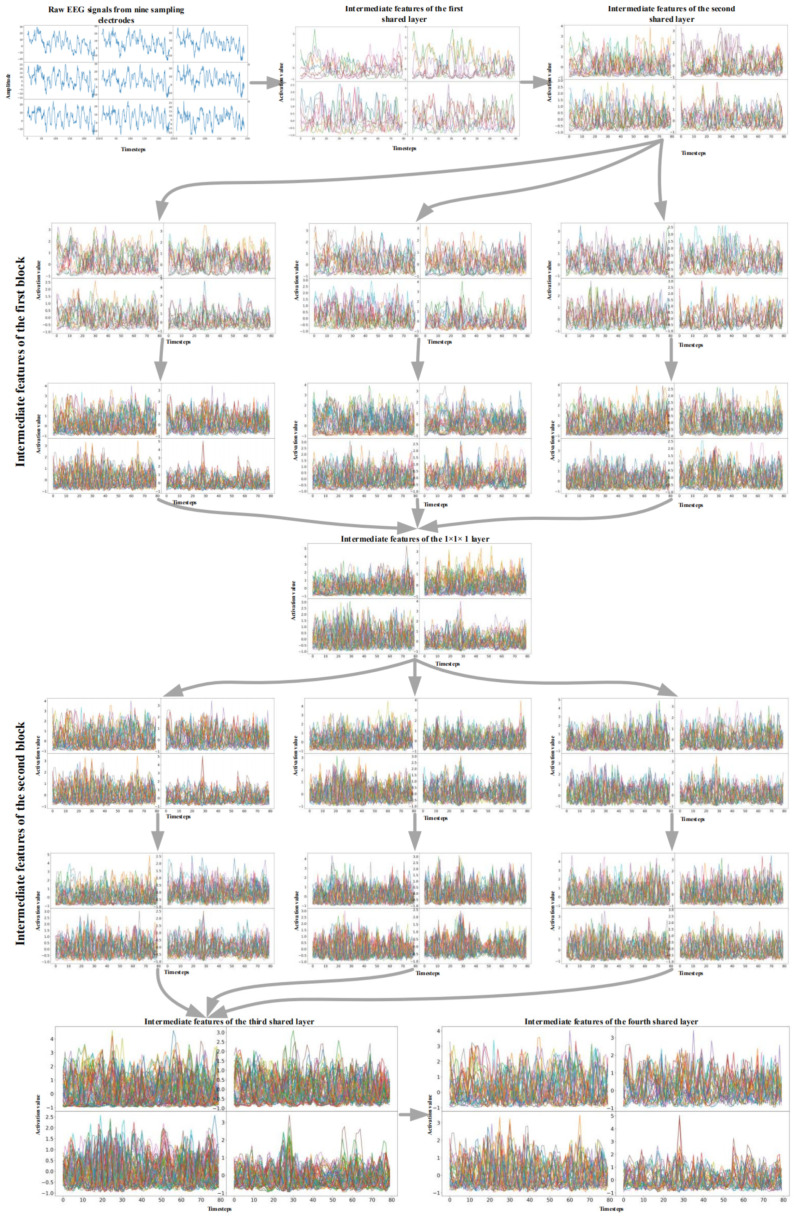
EEG signals from nine C-type sampling electrodes (the first graph) and the features extracted by multi-branch 3D CNN network.

In order to fully display the 3D input and the 4D features constructed by the network, we converted this information into a set of two-dimensional waveforms, as shown in Figure 8. For example, the first graph in Figure 8 shows an EEG signal represented by 3D representation. The distribution of the subgraph represents the spatial distribution of the first two dimensions, that is, the nine electrodes. By a similar method, four series of features obtained by sliding a group of 3D convolution filters along each dimension of the input were converted into four waveform subgraphs. The distribution of the subgraphs represents the spatial distribution of the extracted features. All the subsequent graphs follow the same transformation rules.

Figure 8 shows the analysis results of the visualization of the trained dense M3D CNN for subject 1 for the BCI IV 2a dataset, and we can see several interesting properties from that. Firstly, by observing the input of the raw EEG signals, we found that the EEG signals from different sampling electrodes were slightly different, which is related to the distribution between the electrodes. These differences can be considered as an explanation why the full-set EEG sampling mode can achieve better classification results than fewer-electrode sampling modes (more sampling electrodes means that the EEG signals have more different unique characteristics). 

Secondly, the first convolutional layer reduces the size of the feature map (in both the temporal and spatial dimensions), and it is more like a collection of various waveforms with complementary filters. In the extracted features, almost all the waveform information of the EEG signal is preserved. Thirdly, as the layers become higher, the size of the feature remains unchanged, the extracted features become more abstract, and the visual interpretability becomes worse. In the time domain, the 3D CNN filter begins to provide higher-level concepts with higher activation values, such as certain crest and high-frequency characteristics. This phenomenon can be considered as the extraction process for EEG time-domain features. The localized features that exist on the previous layer begin to disappear, and the uniqueness of features from different spatial locations is more obvious. This phenomenon can be considered as the extraction process for EEG spatial features. As the layers become higher, the extracted features become more complex and unexplainably different from the image field, so it becomes difficult to find the relationship between the represented raw EEG signals and extracted features. Although these representations carry increasingly less information about the visual content of the EEG signals, there is more information related to the classification results, which proves that deepening the network depth can improve the classification accuracy. 

### 4.2. Efficacy of Mean Filling

Zhao et al. [22] first proposed the 3D representation of EEG signals, and then extracted the EEG signal features with a 3D CNN. This 3D presentation is able to preserve both the temporal features of the EEG and the electrode’s spatial features. Their proposed 3D presentation pads the no-electrode point with 0, which has no features of the EEG signals. Here, the proposed 3D presentation pads the points without electrodes with the mean of all the EEG signals, which also contains the features of all the electrode signals. In this manner, the EEG features can be more evident and easier to classify, thereby improving the performance in EEG decoding. Inspired by this method, other padding methods could be considered for future work, as long as the padding methods do not destroy the representative features of the EEG signals. 

### 4.3. Multi-Branch Architecture

In the field of deep learning, many successful methods such as the multi-branch 3D CNN [22] and channel-projection mixed-scale CNN [35] adopt multi-branch architectures. Zhao et al. [22] compared the classification effects of three single-branch 3D CNNs with that of the multi-branch 3D CNN and verified the advantages of a multi-branch framework as compared to the single-branch framework. Our experiments further conclude that with an increase in the branches of dense blocks, the classification effects become better.

**Figure 9:**
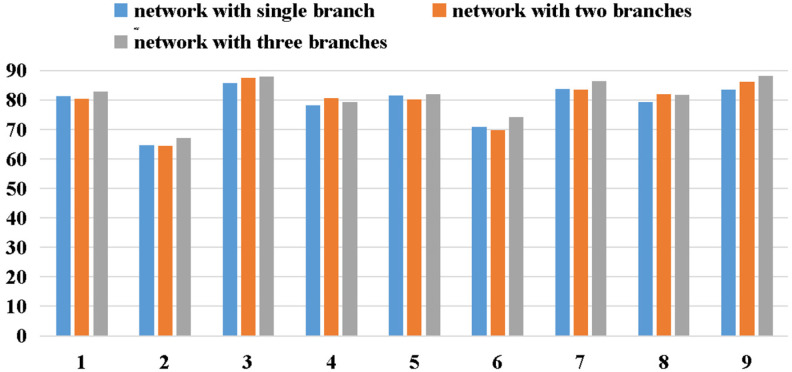
The accuracy comparison of networks with different numbers of branches.

Figure 9 depicts the accuracy comparison of models with different numbers of branches. In general, except for subject 4, the classification accuracy of the network improved significantly with an increase in branches, which proves the effectiveness of the multi-branch network. However, our experiments indicate that with an increase in the number of branches, the parameters increase correspondingly, which results in increased training time and degraded real-time performance. If the computing power is adequate, the classification accuracy can be marginally increased by increasing the number of branches.

### 4.4. Efficacy of Dense Connectivity

**Figure 10:**
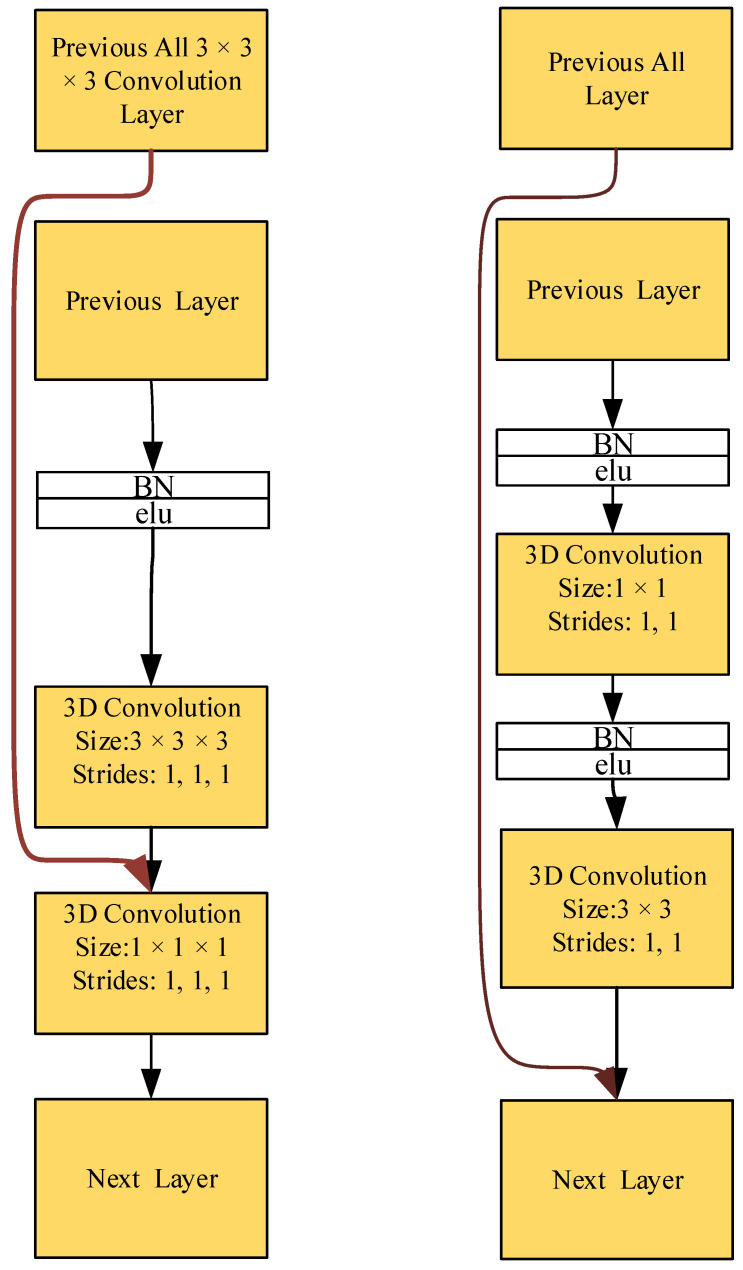
Difference between the proposed dense connection method and the DenseNets. **Left**: the proposed dense connection method. **Right**: dense connection method of DenseNets.

The experimental results in [25] prove that DenseNets utilize the parameters more efficiently than alternative frameworks. Besides, the DenseNet BC with a bottleneck layer is particularly parameter efficient. Owing to the better use of parameters, the dense connectivity can prevent overfitting in a better way. From Figure 10, it can be observed that the proposed dense connectivity is different from that described earlier [25], as that composite function is composed of batch normalization (BN), a ReLU, and a 3 × 3 convolution layer (here, an ELU was taken as the activation function). Moreover, they introduced a 1 × 1 convolution as a bottleneck layer before each 3 × 3 convolution to reduce the number of input feature maps. However, the connection in the proposed method connects the feature maps of the current layer with those produced in all the previous convolution layers (without batch normalization + ELU), similarly, followed by a 1 × 1 × 1 convolution layer.

**Figure 11:**
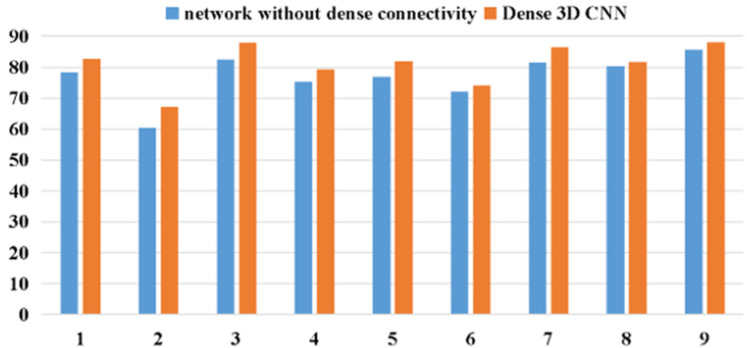
The accuracy comparison of network without dense connections and dense 3D CNN.

As demonstrated in Figure 11, compared to the 3D CNN without dense connections, the proposed dense 3D CNN can effectively improve the classification effect of the framework. It can be concluded that the dense 3D CNN is more competitive than the 3D CNN without dense connections. Due to its dense connectivity, the densely connected 3D CNN enhances the gradient backpropagation, making the model easier to train. Additionally, the dense connectivity enables the network to reuse the features, which can effectively enhance the utilization efficiency for the features in the network.

### 4.5. Network Depth

Researchers have improved the training effect by deepening the depth of the network, such as VGG [23] and ResNet [24]. The experimental results in Table 3 and Table 4 illustrate the depth of our framework as an important factor. In the previous experiment, three different-depth 3D CNNs were designed with one, two, and three dense blocks, respectively. The results in Table 4 suggest that, for the BCI competition IV-2a datasets, the average accuracy of the dense 3D CNN with two dense blocks is higher than that of two other dense 3D CNNs, with one dense block and three dense blocks. This indicates that if the model depth is too shallow, it will not extract features properly, and if the network is too deep, the quantity of the network parameters will be too high to enhance the complexity of the network, implying overfitting and a marginal reduction in the classification effects. Thus, the proposed network with two dense blocks has a relatively appropriate depth for achieving the best classification effects.

For the WAY-EEG-GAL datasets, as shown in Table 4, the average accuracy increased as the network became deeper. This indicates that with an improvement in the model depth, the model can extract the features in a better way. The depth of the model can be further increased to improve the classification accuracy in future work. The different results for the two datasets suggest that different datasets have different optimal model depths. Thus, the depth of the network needs to be changed for different datasets to achieve better classification effects.

Note that, to alter the depth of the network, there are various other ways, such as adding a single convolution layer instead of a dense block to the network and modifying the depth of the dense block so that the network can be changed to accurately obtain the relatively optimal depth. These methods are not discussed in this paper. 

### 4.6. The Influence of Different Initializations

As we know, due to the nature of neural networks, random initializations are required. In this section, we describe the use of three different initialization methods for the BCI competition IV-2a datasets to verify the influence of different parameter initialization methods on the results. The three initialization methods were random initialization with a larger variance (10), large variance (1), small variance (1 × 10^−3^), and smaller variance (1 × 10^−4^), and the variance was 0.01. The results, presented in Table 10, indicate that the kappa values (These kappa values were all obtained from the average kappa values for nine subjects.) with the different initialization methods were approximately equal; this means that different initializations do not affect the results significantly. We think this is mainly due to ADAM (an optimization method), which calculates the updated values of each parameter, so different initialization methods do not affect the results significantly.

### 4.7. Neuroscience and Brain–Computer Interface (BCI)

A brain–computer interface (BCI) is a direct information link between humans and external devices. A brain–computer interface system takes EEG signals as input signals, and then, through signal processing, it can identify a human’s intention and, finally, convert the human’s thinking activity into a command signal, which can realize the control of external devices and communication with the outside world; furthermore, it can also input information into the brain through electrical stimulation and interact with the brain. As a new, complex, and interdisciplinary technology, brain–computer interface technology is widely used in many fields, and its future prospects are expected. Recently, neuroscience, the basis of the brain–computer interface, has made great progress, which is directly promoting the development of brain–computer interfaces. The research into and applications of neuroscience and brain–computer interfaces will surely benefit many people, especially motor-disabled people. The EEG signal-decoding algorithm applied in BCIs will also become very important. Our algorithm is simple and efficient, has good classification effects and good robustness, and can be widely used in BCI applications.

## 5. Conclusions

In the discussed proposal, an improved 3D representation method is mentioned, which fully utilizes the features of EEG signals in the 3D presentation. Furthermore, based on that approach, a densely connected multi-branch 3D CNN was designed for motor imagery EEG classification tasks. The densely connected multi-branch 3D CNN can effectively extract EEG features to achieve good performance in motor-imagery-classification tasks due to its deep structure and dense connectivity. The classification effect for the BCI IV 2a datasets and the WAY-EEG-GAL datasets suggests that the proposed method has better performance as compared to the state-of-the-art multi-branch 3D CNN. Furthermore, the proposed method is user-friendly and can be applied to other MI-classification tasks as an effective method.

## Figures and Tables

**Table 1:** Overall structure parameter of dense M3D CNN.

Layer Name	Size	Strides	Filters
Conv1	3 × 3 × 3	2 × 2 × 2	8
Conv	3 × 3 × 3	1 × 1 × 1	16
	1 × 1 × 1	1 × 1 × 1	16
Block1	Dense multi-branch 3D CNN block		32
Block2	Dense multi-branch 3D CNN block		96
Conv	3 × 3 × 3	1 × 1 × 1	96
	1 × 1 × 1	1 × 1 × 1	96
Conv	3 × 3 × 3	1 × 1 × 1	96
	1 × 1 × 1	1 × 1 × 1	96
Dense (Dropout rate = 0.5)	256		
Dense (Dropout rate = 0.5)	64		
Dense	Different MI classes		

Conv means 3 × 3 × 3 3D convolution layer + bottleneck layers + batch normalization (BN) + ELU.

**Table 2:** The numbers of training data of four motion stages obtained by cropping strategy.

Subject ID	C1	C2	C3	C4
S1	5512	196,238	5560	5295
S2	52,032	276,729	6327	14,673
S3	6065	186,403	5487	5530
S4	6278	201,843	8543	8792
S5	27,890	228,718	6202	7210
S6	26,462	233,637	6147	14,712
S7	78,849	239,767	21,997	26,298
S8	10,791	217,182	5676	6036
S9	27,349	210,387	7009	14,817
S10	25,707	251,869	8154	9750
S11	56,070	233,336	7971	19,627
S12	51,158	247,668	19,423	41,810

**Table 3:** Comparison of cross-validation results for four different networks for the BCI IV-2a datasets.

Subject	Multi-Branch 3D CNN	Dense M3D CNN1	Dense M3D CNN2	Dense M3D CNN3
1	77.397	81.333	82.894	77.500
2	60.140	63.156	67.336	60.254
3	82.927	88.019	88.117	87.889
4	72.288	80.261	79.444	76.556
5	75.836	80.778	82.117	83.483
6	68.988	72.444	74.285	72.536
7	76.036	84.667	86.563	85.601
8	76.855	82.325	81.918	81.567
9	84.665	87.556	88.333	86.588
Mean	75.015	80.060	81.223	79.108
Standard deviation	7.344	7.841	6.845	8.719
*p*-value (multi-branch 3D CNN)	-	4.07 × 10^−5^	6.16 × 10^−6^	2.37 × 10^−3^
*p*-value (dense M3D CNN1)	4.07 × 10^−5^	-	2.41 × 10^−2^	0.114
*p*-value (dense M3D CNN2)	6.16 × 10^−6^	2.41 × 10^−2^	-	2.19 × 10^−2^
*p*-value (dense M3D CNN3)	2.37 × 10^−3^	0.114	2.19 × 10^−2^	-

*p*-value (“NETWORK”) means the significance of the difference (*p*-value) between different networks’ performance.

**Table 4:** Comparison of cross-validation results for four different networks for the WAY-EEG-GAL datasets.

Experiment	Class	Multi-Branch 3D CNN	Dense M3D CNN1	Dense M3D CNN2	Dense M3D CNN2
C1 and C2	C1	55.623	75.565	73.479	74.281
	C2	83.557	79.576	80.407	81.217
C2 and C3	C2	94.305	89.281	90.885	90.541
	C3	51.584	64.748	66.438	65.854
C3 and C4	C3	70.259	77.517	75.542	76.853
	C4	68.746	71.882	73.523	73.015
Mean		70.679	76.428	76.712	76.960
Standard deviation		16.243	8.175	8.271	8.351
*p*-value (multi-branch 3D CNN)		-	9.04 × 10^−3^	7.82 × 10^−3^	7.22 × 10^−3^
*p*-value (dense M3D CNN1)		9.04 × 10^−3^	-	0.359	0.163
*p*-value (dense M3D CNN2)		7.82 × 10^−3^	0.359	-	0.246
*p*-value (dense M3D CNN3)		7.22 × 10^−3^	0.163	0.246	-

*p*-value (“NETWORK”) means the significance of the difference (*p*-value) between different networks’ performance.

**Table 5:** Comparison of cross-validation results for training on two different networks with different 3D presentations.

Subject	Multi-Branch 3D CNN	Multi-Branch 3D CNN *	Dense M3D CNN2	Dense M3D CNN2 *
1	77.397	77.725	81.556	82.894
2	60.140	61.578	66.222	67.336
3	82.927	84.751	87.778	88.117
4	72.288	73.892	79.658	79.444
5	75.836	78.335	81.889	82.117
6	68.988	72.414	74.556	74.285
7	76.036	78.667	85.667	86.563
8	76.855	79.287	82.556	81.918
9	84.665	85.222	88.556	88.333
Mean	75.015	76.875	80.938	81.223
Standard deviation	7.344	7.127	6.982	6.845

* indicates that the network was trained with our proposed improved 3D presentation.

**Table 6:** Comparison of cross-validation results for training on network without dense connection with results for the proposed network.

Subject	M3D CNN2	Dense M3D CNN2
1	78.424	82.894
2	60.473	67.336
3	82.561	88.117
4	75.549	79.444
5	76.982	82.117
6	72.215	74.285
7	81.617	86.563
8	80.364	81.918
9	85.842	88.333
Mean	77.114	81.223
Standard deviation	7.431	6.845
*p*-value (M3D CNN2)	-	5.71 × 10^−5^
*p*-value (dense M3D CNN2)	5.71 × 10^−5^	-

*p*-value (“NETWORK”) means the significance of the difference (*p*-value) between different networks’ performance.

**Table 7:** Comparison of cross-validation results for training on networks with different numbers of branches in dense block.

Subject	Dense 3D CNN *	Dense 3D CNN **	Dense M3D CNN
1	81.33	80.556	82.894
2	64.731	64.518	67.336
3	85.956	87.593	88.117
4	78.289	80.847	79.444
5	81.634	80.349	82.117
6	70.953	69.812	74.285
7	83.913	83.667	86.563
8	79.385	82.014	81.918
9	83.749	86.382	88.333
Mean	78.895	79.526	81.223
Standard deviation	6.864	7.571	6.845

* indicates that there is one branch in dense block; the ** means there are two branches.

**Table 8:** Comparison of the kappa values for different sampling types and the state of the art.

Subject ID	Full-Set	A-Type	B-Type	C-Type	FBCSP	C2CM	Multi-Branch 3D CNN
1	0.775	0.643	0.635	0.710	0.68	0.833	0.699
2	0.543	0.409	0.416	0.440	0.42	0.537	0.459
3	0.824	0.750	0.781	0.698	0.75	0.870	0.788
4	0.604	0.673	0.538	0.541	0.48	0.556	0.594
5	0.777	0.638	0.486	0.584	0.40	0.5	0.647
6	0.591	0.556	0.490	0.421	0.27	0.273	0.538
7	0.837	0.701	0.727	0.651	0.77	0.861	0.653
8	0.672	0.605	0.608	0.584	0.76	0.778	0.702
9	0.870	0.790	0.757	0.845	0.61	0.727	0.713
Mean	0.721	0.641	0.604	0.608	0.571	0.659	0.644
Standard deviation	0.121	0.112	0.131	0.135	0.206	0.204	0.100
*p*-value (full-set)	-	3.56 × 10^−3^	6.38 × 10^−4^	2.27 × 10^−4^	7.06 × 10^−3^	9.54 × 10^−3^	5.06 × 10^−3^
*p*-value (A-type)	3.56 × 10^−3^	-	7.30 × 10^−2^	2.24 × 10^−2^	1.09 × 10^−2^	0.37	0.442
*p*-value (B-type)	6.38 × 10^−4^	7.30× 10^−2^	-	0.436	0.199	0.115	6.55 × 10^−2^
*p*-value (C-type)	2.27 × 10^−4^	2.24 × 10^−2^	0.436	-	0.222	0.151	0.106
*p*-value (FBCSP)	7.06 × 10^−3^	1.09 × 10^−2^	0.199	0.222	-	3.28 × 10^−4^	6.27 × 10^−3^
*p*-value (C2CM)	7.06 × 10^−3^	0.37	0.115	0.151	3.28 × 10^−4^	-	0.377
*p*-value (multi-branch 3D CNN)	5.06 × 10^−3^	0.442	6.55 × 10^−2^	0.106	6.27 × 10^−3^	0.377	-

*p*-value (“METHOD”) means the significance of the difference (*p*-value) between different methods’ performance.

**Table 9:** Training time and testing time.

	Training Time	Testing Time
Full-set	00:33:05	00:00:21
A-type	00:27:57	00:00:17
B-type	00:27:41	00:00:17
C-type	00:28:05	00:00:17
Dense 3D CNN *	00:23:26	00:00:15
Dense 3D CNN **	00:29:41	00:00:19

The * indicates that there is one branch in the dense block, and the ** suggests the presence of two branches.

**Table 10:** The kappa values with different initialization methods.

Variance	1 × 10^−4^	1 × 10^−3^	0.01	1	10
Average kappa	0.720	0.715	0.719	0.722	0.717
